# The influence of high and low levels of estrogen on diurnal urine regulation in young women

**DOI:** 10.1186/1471-2490-8-16

**Published:** 2008-11-19

**Authors:** Charlotte Graugaard-Jensen, Gitte M Hvistendahl, Jørgen Frøkiær, Peter Bie, Jens Christian Djurhuus

**Affiliations:** 1 The Institute of Clinical Medicine, University Hospital of Aarhus, Skejby Sygehus, 8200 Aarhus N, Denmark; 2 Department of Urology, University Hospital of Aarhus, Skejby Sygehus, 8200 Aarhus N, Denmark; 3 Department of Clinical Physiology and Nuclear Medicine, University Hospital of Aarhus, Skejby Sygehus, 8200 Aarhus N, Denmark; 4 Department of Physiology & Pharmacology, University of Southern Denmark, 5000 Odense, Denmark

## Abstract

**Background:**

Sex hormones have a pronounced effect on arginine vasopressin (AVP), and therefore on the diurnal water homeostasis. Low and high levels of plasma-estradiol as seen in the follicular phase of the menstrual cycle may therefore alter the diurnal regulation of urine production. Furthermore the structural resemblance of oxytocin to vasopressin has led to speculations about the possible antidiuretic properties of oxytocin under normal physiological conditions. To elucidate the influence of high and low p-estradiol on the regulation of the diurnal urine production, 15 normal menstruating women (21–33 y) underwent two circadian in-patient investigations, both situated in follicular phase.

**Methods:**

Admitting the participants solely in the follicular phase resulted in high and low plasma-estradiol whereas plasma-progesterone was similar. Urine and blood samples were taken at predetermined time points to determine plasma AVP, plasma oxytocin, plasma aldosterone, plasma natriuretic peptide (ANP), urinary solute excretions, and urinary excretions of prostaglandin E2 (PGE-2) and aquaporin-2 (AQP-2). Blood pressure was measured every hour.

**Results:**

Plasma AVP, plasma aldosterone and plasma ANP were unaffected by the different levels of estradiol. All had marked circadian variations whereas oxytocin did not display any circadian rhythm. High estradiol resulted in lower p-osmolality and p-sodium reflecting the downward resetting of the osmoreceptors. Oxytocin did not correlate with either diuresis or urine osmolality. The diurnal urine production was similar in the two groups as were urine osmolality, excretion of PGE-2 and AQP-2. AQP-2 does not have a circadian rhythm and is not significantly correlated to either AVP or oxytocin under normal physiological conditions.

**Conclusion:**

High and low level of estradiol has no influence on the circadian rhythm of AVP or the subsequent urine production. High p-estradiol resets the osmoreceptors for AVP release. Furthermore it appears that oxytocin under normal physiological conditions do not contribute to the overall antidiuretic effect.

## Background

The transition from day to night lead to a pronounced decrease in diuresis and a reduction in excretion of electrolytes in both sexes. The secretion of AVP, one of the most important hormones in the human water homeostasis increases at night. The diurnal rhythm of AVP is most pronounced in tween-agers [1], whereas the rhythm in adolescents is attenuated [2]. A diurnal rhythm all though less marked exist in adult males as well [3-6]. Circulating AVP is influenced by ovarian steroid blood levels and various studies have shown changing AVP concentration during the normal menstrual cycle in women [7,8]. Other studies have failed to confirm any variation [9-12]. Estrogen unopposed to progesterone given to postmenopausal women will increase AVP, whereas the combination of estrogen and progesterone will decrease AVP [13-16]. Only a few circadian studies of AVP have been conducted in females [8,17,18]. One study in premenopausal women has demonstrated both increased basal and nocturnal concentration of AVP in midfollicular phase of the menstrual cycle [8]. Another study has shown decreased circadian rhythm in young women compared to young age-matched men, but are without control for menstrual status in the young women [18]. Stimulated release of vasopressin is influenced by sex hormones as well. Baseline plasma-osmolality and sodium are decreased in high estrogen states due to a resetting of the osmoreceptors for thirst and AVP release [9,10,12,19-21]. In spite of the lower plasma osmolality, AVP secretion persists, reducing clearance of free water and maintaining slightly hypotonic plasma.

The structural resemblance of oxytocin to AVP has led to speculations of a possible additive antidiuretic effect of oxytocin. Exogenous oxytocin in doses above the normal range is clearly antidiuretic [22,23], and since estrogen increases the concentration of oxytocin [8,24,25], it is obvious to compare the antidiuretic effect of oxytocin in different estrogen states. Observations concerning the diurnal rhythm of oxytocin over the course of a normal menstrual cycle are however sparse [8] and the role of oxytocin as an antidiuretic hormone under normal physiologic conditions remains to be clarified. Since the hormones involved in the diurnal urine regulation one way or another all are influenced by the female sex-hormones, it seems reasonable to test the hypothesis that different levels of estrogen alters the diurnal regulation of urine production. To identify the effect of different concentrations of estrogen on the diurnal urine regulation, the volunteers were admitted twice and solely in the follicular phase, in which progesterone is low. We hypothesized that: 1) AVP and the nocturnal peak are augmented in women with high endogenous estrogen compared to women with low endogenous estrogen. The urine production will subsequently be decreased and urine osmolality increased in the high estrogen-state. 2) Oxytocin would be enhanced when endogenous estrogen rises preovulatory and may subsequently affect the diurnal urine regulation.

## Methods

Fifteen healthy non-smoking women were recruited by advertisement at the University of Aarhus. The participants should have a normal reproductive history and the natural cycling women should have regular menstrual cycles (26–33 days). They should not have nocturia or any other urological complaints, no previous surgery, no history of renal, cardiac or hepatic disease and a normal physical examination (gynecologic examination included), normal office blood pressure, normal blood chemistry, normal urine-analysis (no bacteuria, proteinuria or glucosuria), normal uroflowmetry and no post void residual volume assessed by ultrasound estimation.

No medication should be taken for at least 1 month before and during the investigation. All participants volunteered to the study after information of its nature and purpose, which conformed to the guidelines, contained in the Declaration of Helsinki, with a prior approval by the Local Ethics Committee in Aarhus County and the Danish Data Protection Agency.

In the preceding cycle the participants twice completed a 3-day frequency/volume chart (FVC). Time and volume (ml) of each micturition and of each fluid intake were noted. Intake of fluid and food was *ad libitum *and the subjects were allowed to ambulate at will. No restrictions were instigated regarding type of foods or fluids. The participants were asked not to alter their habits during the measuring period. The time of rising and the bedtime was noted.

The participants were admitted twice. To plan the dates for the two admissions, data from the three latest cycles in each participant were used. Admissions took place in the follicular phase in which progesterone remains low. The first admission being in the middle of the follicular phase representing a low estrogen phase and the second 5 days later just before estimated time of ovulation representing a high estrogen phase. Two days prior to the admissions, the participants were fluid preconditioned to 30 ml/kg body weight/24 h. The participants were told to refrain from alcohol and caffeinated beverages and heavy physical activities 24 h before the admission. The participants were then admitted to the hospital for 25 hours.

Upon arrival an intravenous catheter was inserted in the antecubital vein in the non-dominant arm. Blood-samples were obtained at 8, 14, 20, 23, 2, 5, and 8 hours. A total amount of 210 ml blood was drawn. The blood samples were replaced by isotonic saline.

Participants were seated in a chair 30 min before blood sampling during the day (0800 to 2300 h). During the night (2300 to 0800 h) samples were taken in the supine position. Blood samples were centrifuged at +4°C and plasma samples were stored at -80°C unless immediately analyzed. Parameters analyzed were atrial natriuretic peptide (ANP), AVP, estradiol, oxytocin, and prolactin, progesterone, testosterone, FSH and LH. Creatinine (Pcr), packed cell volume (PCV), potassium (PK), osmolality (Posm), and sodium (PNa) were analyzed as well.

Blood pressures were monitored by cuff every hour with an automatic ambulatory blood pressure monitor (ABP Monitor 90207 ™ SpaceLabs Medical, Inc, Redmond, WA, USA).

Urine was collected every three hours from 0800 to 2300 hours. Night-time urine production was represented by one sample (2300-0800 h). Urine volume (Uvol) and the concentration of creatinine (Ucr), osmolality (Uosm), potassium (UK), and sodium (UNa) as well as prostaglandin E2 (PGE-2) and aquaporin-2 (AQP-2) were measured.

During admission a total fluid intake of 30 ml/kg body weight/24 h of tap water, distributed equally during the day was allowed. Meals with known contents of sodium (3 mmol/kg/day), potassium, calcium, and calories were served at 0800, 1200, and 1800 hours.

### Blood and urine analyses

Plasma and urine concentration of sodium, potassium and creatinine, PCV, plasma concentration of estradiol, progesterone, prolactin, testosterone, FSH, and LH were measured using routine procedures. Plasma and urine osmolality were measured using the freezing point depression method (Advanced™ Osmometer model 3900, Advanced Instruments, Norwood, MA, USA).

### Analyses of hormones

AVP was determined in plasma by radioimmunoassay (RIA)[26], using a highly specific AVP antibody (AB3096) as previously described by [27]. AVP was extracted from plasma using Sep-Pak^® ^Plus C18 extraction cartridge (Waters Corporation, Milford, MA, USA). The detection limit was 0.10 pg/ml plasma and the intra- and inter-assay coefficients of variation were 7.7% and 10.6%, respectively.

ANP was measured in plasma following extraction on Sep-Pak^® ^Plus C_18 _extraction cartridge (Waters Corporation, Milford, MA, USA) by means of a RIA using a sheep ANP specific antibody (1023BANI1, Euro-diagnostica, Arnhem, The Netherlands). The detection level was 3.5 pg/ml, whereas inter- and intra-assay coefficients of variation were 11.6% and 8.6% respectively.

Aldosterone was measured without extraction by means of a RIA using a rabbit antibody (DSL-8600, Diagnostic Systems Laboratories, Inc., Webster, Texas, USA). Detection level 7.64 pg/ml. Inter- and intra-assay was 8.2% and 3.9% respectively.

Oxytocin was measured in plasma following extraction on Sep-Pak^® ^Plus C_18 _extraction cartridge (Waters Corporation, Milford, MA, USA) by RIA using an oxytocin-specific rabbit-antibody kindly provided by Dr. M. Morris, Dayton, OH, USA. The detection level was 0.9 pg/ml, whereas inter- and intra-assay coefficients of variation were 11.0% and 7.5% respectively.

### Urine analyses

A commercial enzyme immunoassay kit (Amersham Pharmacia Biotech, Little Chalfont, UK) was used to determine the PGE_2 _excretion in the urine. The analysis was performed without prior extraction. Inter-assay and intra-assay were approximately 13% and 9%. The detection limit was 40 pg/ml urine.

Urinary AQP2 was measured by radioimmunoassay using a modification of previous established methods [28-30]. Detection limit was 16 pg/ml urine.

### Calculations

Based on the measurements, excretion rates (E) and clearances (C) were calculated for electrolytes, creatinine and osmoles (osm) using standard formulas. Glomerular filtration rate (GFR) was estimated from creatinine clearance (Ccr). Fractional excretions (FE_x_) was calculated as C_x_/GFR*100. Solute free water reabsorption (T_C _H_2_O) is Cosmol-Uvol.

### Statistical analyses

To compensate for the variability of body weight in the study population urinary excretion rates are expressed per kg body weight. Participants who had a mean p-estradiol below 0.5 nmol/l in the first admission and at least a 75% increase in p-estradiol to the second admission were included in the statistical analysis. To detect effects in p-AVP of approximately 0.20 or greater, the sample size was estimated to 7 using pair wise comparisons. A = 0.05 and β = 0.2.

To compare the evolution over time for the two groups, we used a mixed effect ANOVA with a subject specific random effect and using time and group as fixed effects, as well as their interaction if this was statistically significant (Wald's test). When quantifying the day-night differences we created an indicator variable (day at time points 0800, 1400, 2000, and 2300 and night at time points 0200, 0500, and 0800) and used this variable in place of time in the mixed effect model. When appropriate the data were log-transformed to fit the statistical model. These data are presented as geometric mean with normal 95% confidence intervals. Regarding the home recordings, 24 h, day and night time parameters were calculated and differences between day and night were compared with Student's t tests. Correlations were calculated using Pearson's correlation. When correlating parameters obtained from both blood and urine, only simultaneously taken blood samples and urine samples were correlated.

The results are presented as mean ± SD. Non-detectable values were set to equal half the detection level. One of the participants is excluded from the analysis of PGE-2, because she had three extreme outliers of unknown origin in her second admission. Results were considered significant at p < 0.05.

## Results

Seven of the admitted women were excluded prior to the statistical handling due to either low or medium estrogen-levels in both admissions; this assumed to be due to irregularity in the menstrual cycle at the time of investigation.

The analysis thus includes 8 women (mean age 25.0 years, range 23–26 years; bodyweight 64.6 kg, range 56–78 kg) who fulfilled the criteria of a mean p-estradiol below 0.5 nmol/l in the first admission and at least a 75% increase in p-estradiol to the second admission.

### Home-recordings

During the home-recording period, all of the 8 included participants completed the FVC for 2 times 3 days and nights. There was no difference in any of the measured parameters between the two 3-day periods. In total 2 nocturia nights and 46 no-nocturia nights were recorded. The 24 h fluid-intakes at home were 30.3 ± 6.2 ml/kg. The fluid intake at home did not differ from the fluid intake in the inpatient studies. Both 24 h urine volume and daytime urine volume during admissions 1 and 2 were significantly higher than in the home-recordings. Day/night ratio was similar in both admissions and in the home-recordings (Table 1).

**Table 1:** Fluid intake and urine production at home assessed from a frequency volume chart (FVC) and during the in-patient studies (1. and 2. admission)

			Mean ± SD				
			
	Registered days and nights	Nocturia nights	Fluid intake (ml/kg)	24 h urine production (ml/kg)	Day time urine production (ml/kg)	Night time urine production (ml/kg)	Day/night ratio
Home	48	2	30.27 ± 6.19	26.93 ± 6.00	22.08 ± 7.74	5.98 ± 1.00**	3.98 ± 2.04

1. admission	8	1	30	38.42 ± 8.80^¶^	31.28 ± 9.14^¶^	7.14 ± 2.43***	4.86 ± 2.43

2. admission	8	1	30	40.88 ± 6.09^¶¶^	33.12 ± 8.83^¶¶^	7.76 ± 5.42**	5.92 ± 3.81

Values are mean ± SD. ** day/night p < 0.01, *** day/night p < 0.001, ^¶ ^Home/in-patient p > 0.05, ^¶¶ ^Home/in-patient p > 0.01

### Urine excretions

24-hour and night time urine volume were similar in the two admissions (Table 2) and a statistically significant decrease in urine output from day to night was registered. Two participants experienced nocturia in one of their admissions (at 0500 h and midnight, respectively) accompanied by a decreased day/night ratio.

**Table 2:** Renal clearances and urinary excretions during the two admissions (1 and 2)

	Mean ± SD					
	
	1. admisission			2. admission		
	
	Day	Night	24 h	Day	Night	24 h
Uflow(ml/kg/h)	2.13 ± 0.58	0.76 ± 0.27***	1.62 ± 0.34	2.22 ± 0.55	0.83 ± 0.56***	1.70 ± 0.23

Uosm (mosm/kg)	431 ± 112	550 ± 126**	476 ± 90	382 ± 111	549 ± 181**	445 ± 80

E_Na _(mmol/kg/h)	0.16 ± 0.05	0.07 ± 0.02***	0.13 ± 0.03	0.14 ± 0.04	0.07 ± 0.05***	0.12 ± 0.03

C_Na _(ml/kg/h)	1.19 ± 0.35	0.45 ± 0.14***	0.91 ± 0.20	1.04 ± 0.27	0.46 ± 0.33***	0.83 ± 0.23

FE_Na _(%)	0.92 ± 0.33	0.36 ± 0.11***	0.71 ± 0.20	0.80 ± 0.23	0.37 ± 0.32***	0.64 ± 0.21

E_K _(mmol/kg/h)	0.06 ± 0.02	0.02 ± 0.01***	0.04 ± 0.01	0.06 ± 0.02	0.02 ± 0.01***	0.04 ± 0.01

C_K _(ml/kg/h)	13.92 ± 3.36	4.86 ± 1.84***	10.52 ± 2.35	14.72 ± 5.34	4.66 ± 2.46***	10.95 ± 3.04

FE_K _(%)	11.51 ± 3.44	3.83 ± 1.36***	8.63 ± 2.31	11.51 ± 4.05	4.16 ± 1.71***	8.75 ± 2.48

E_osm _(mmol/kg/h)	0.76 ± 0.11	0.45 ± 0.10***	0.64 ± 0.08	0.72 ± 0.10	0.45 ± 0.16***	0.62 ± 0.10

C_osm _(mosm/kg/h)	2.68 ± 0.37	1.40 ± 0.31***	2.20 ± 0.25	2.54 ± 0.38	1.43 ± 0.53***	2.12 ± 0.36

C_Cr _(ml/kg/h)	132.45 ± 16.29	126.99 ± 17.87	130.30 ± 19.72	134.43 ± 18.35	133.20 ± 21.74	133.97 ± 17.61

TC_H2O _(ml/kg/h)	0.55 ± 0.42	0.64 ± 0.27	0.58 ± 0.30	0.32 ± 0.60	0.59 ± 0.33	0.42 ± 0.41

E_PGE2 _(ng/kg/h)	2.96 ± 0.66	1.87 ± 0.69***	2.47 ± 0.74	3.18 ± 0.50	2.08 ± 0.47**	2.64 ± 0.28

E_AQP2 _(pg/ml/h)	0.19 ± 0.11	0.17 ± 0.13	0.18 ± 0.10	0.18 ± 0.08	0.17 ± 0.06	0.17 ± 0.07

Values are mean ± SD. See Methods for calculations. Day/night differences: ** day/night p < 0.01 and *** day/night p < 0.001

**Figure 1:**
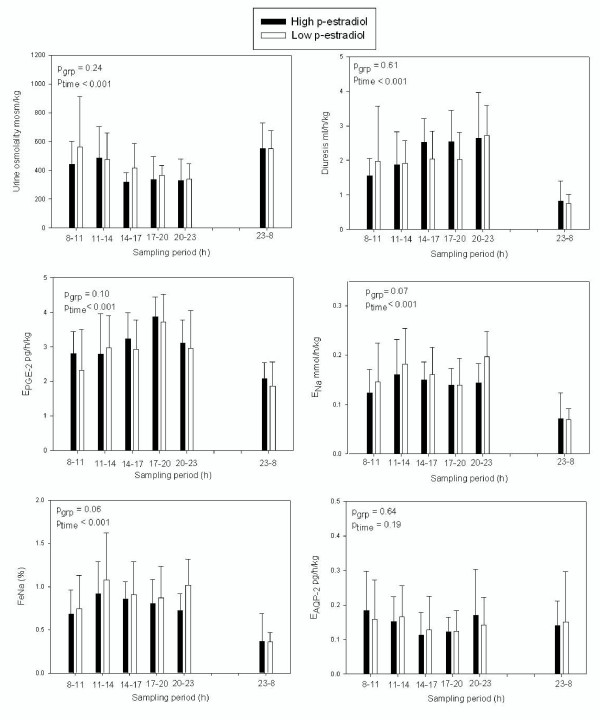
**Diurnal rhythm of urine osmolality, diuresis, excretion rates of PGE-2, AQP-2 and sodium in women with low (white) or high (black) p-estradiol. **Bars are mean ± SD.

Urine-production peaked between 2000 and 2300 hours and the lowest diuresis was seen at night (p < 0.001) (Fig. 1).

In both admissions a night time increase in urine osmolality was seen (p < 0.01). No difference was found between the two admissions for the overall 24 h excretion of sodium, potassium, osmoles, AQP-2 or PGE_2 _(Table 2). Except for AQP-2, there was a marked decrease in excretion during night time in both admissions. Similar results were obtained for C_Na_, C_K_, and C_osm_. There was, however a tendency towards decreased clearance of sodium, excretion of sodium and fractional excretion of sodium in women with high estrogen, but it did not quite reach statistical significance (p = 0.080, p = 0.063 and p = 0.074, Fig. 1+2).

**Figure 2:**
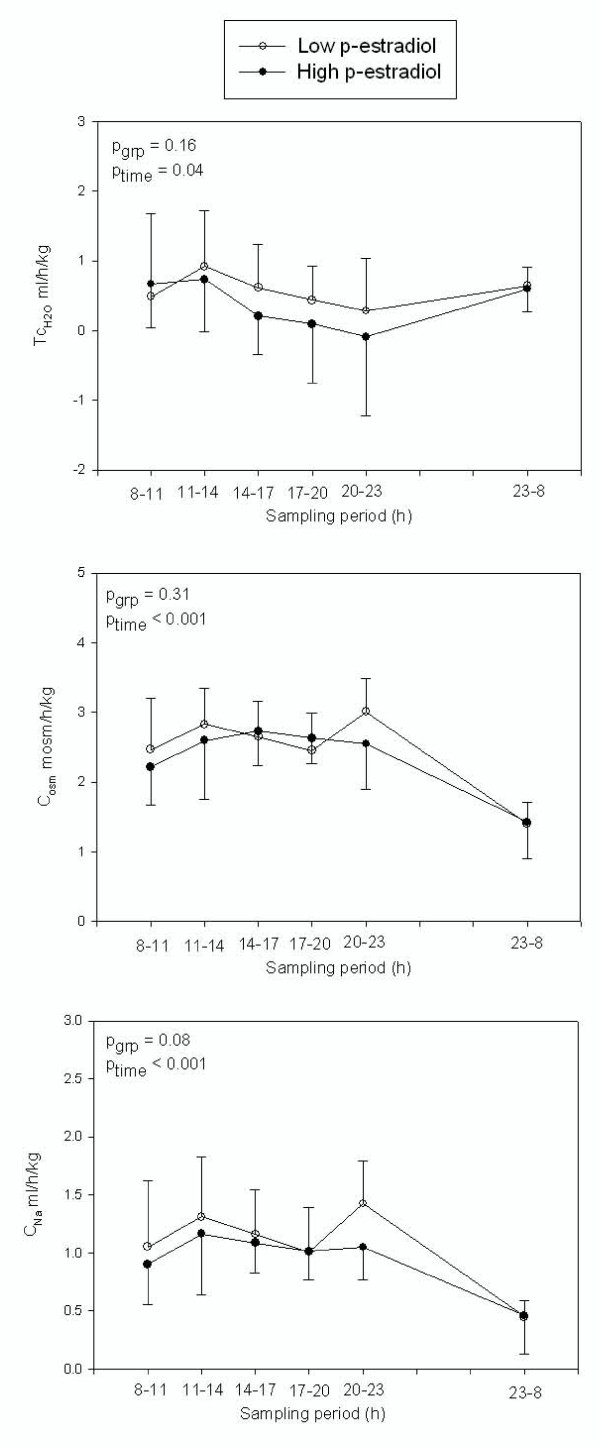
**Diurnal variation of clearance of sodium and osmoles, and solute free water reabsorption in natural cycling women with low (white) and high (black) p-estradiol.** Values are mean ± SD.

We found no diurnal variation in glomerular filtration rate estimated by creatinine clearance (C_Cr_) or in solute free water reabsorption (T_C_). There was no change in GFR or solute free water reabsorption between the two admissions (p = 0.52 and p = 0.16, respectively, Fig. 2).

### Measurements of plasma variables

**Figure 3:**
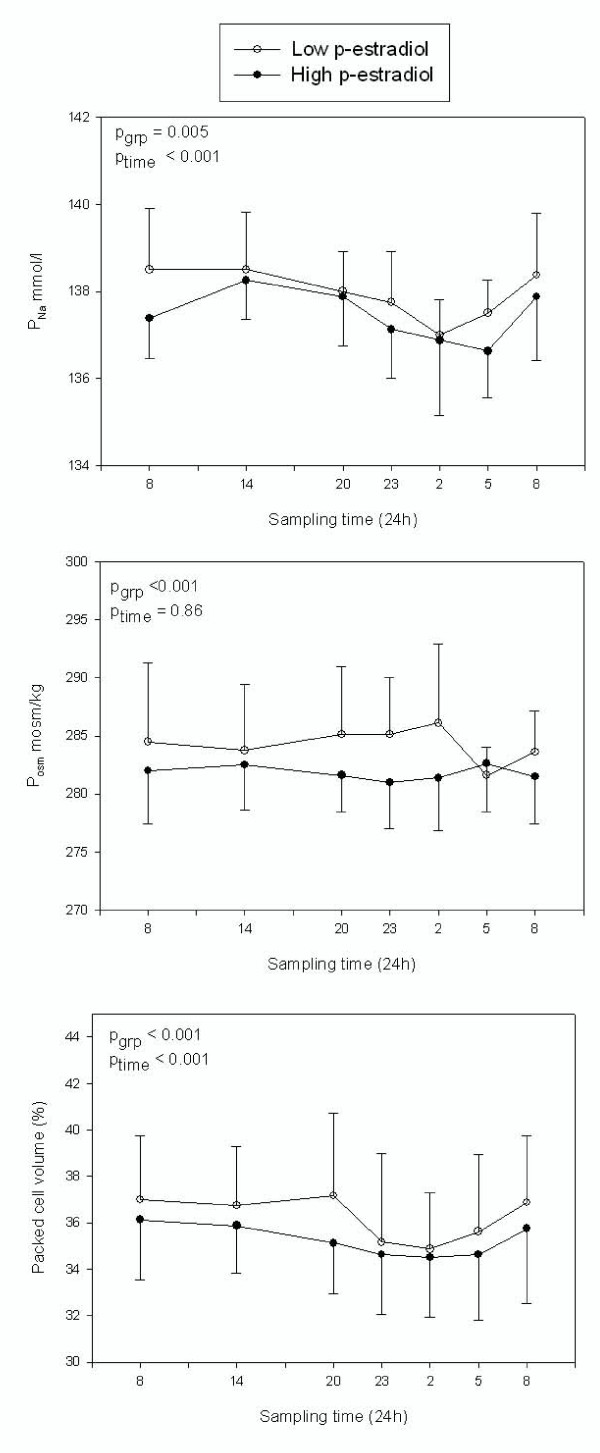
**P-sodium, p-osmolality and packed cell volume in low (white) and high (black) estrogen states.** Values are mean ± SD.

Plasma osmolality had no diurnal variation, but was lower in the second admission (high estrogen). The difference between p-osmolality in the two admissions: 2.46 ± 0.62 mosm/kg, (95%: 1.23–3.70, p < 0.001). Plasma sodium concentration showed significant diurnal variation with a reduction in concentration at night (p < 0.001). Women with high estrogen (2. admission) had significantly lower plasma sodium, the difference between p-sodium in the two admissions: 0.51 ± 0.18 mmol/l (95%: 0.15–0.87, p = 0.005), figure 3. Plasma creatinine had in both admissions a diurnal variation with an increase of 7.06 ± 2.31 μmol/l, (95%: 2.53–11.59, p = 0.002) at 2000 hours.

There was a small, but consistent reduction in packed cell volume between the two admissions: 1.03 ± 0.28%, (95%: 0.48–1.58, p < 0.001). Furthermore, we observed a minimal night time decrease (1.34 ± 0.41%, (95% CI: 0.53–2.14, p < 0.001) compared to mean daytime values.

### Hormones

**Figure 4:**
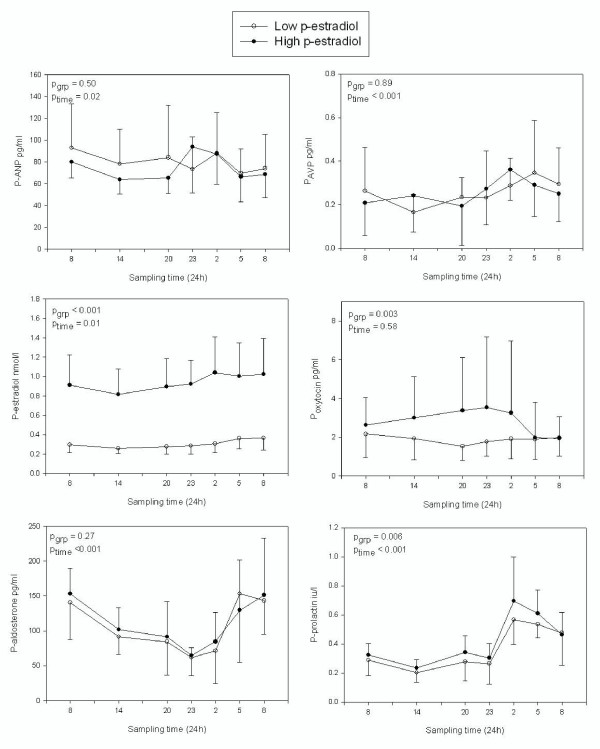
**Circadian rhythm of the hormones ANP, AVP, oxytocin, aldosterone and prolactin in women with low and high p-estradiol. **The obtained difference in p-estradiol between low and high states is shown in the graph as well. Values are mean ± SD.

Mean p-estradiol-concentrations during the two admissions are presented in table 3. P-AVP and p-ANP were unaffected by phase of menstrual cycle. For both hormones we found a significant circadian rhythm (Fig. 4). AVP increased at night. The ratio between geometric mean day time and mean night time was 1.46 ± 0.15 pg/ml, p < 0.001, whereas ANP did not have a nocturnal increase (p = 0.52). For prolactin we found a marked circadian variation with a consistent peak at 0200 hours (p < 0.001). A significant difference was found between the groups as well; as prolactin levels were higher in women with high estrogen (p = 0.006). Plasma oxytocin was increased along with estradiol but showed no circadian rhythm (p = 0.58). A clear diurnal variation was also evident for p-aldosterone levels. The plasma levels of the hormone markedly declined towards the evening and the first hours of the night to continuously rise hereafter until the early morning. There were no differences in the concentration of progesterone; 3.44 ± 2.22 vs. 3.45 ± 1.01 nmol/l (p = 0.80) or testosterone 1.50 ± 0.54 vs. 1.64 ± 0.62 nmol/l (p = 0.30) between the two admissions.

**Table 3:** Plasma-estradiol concentration in the two admissions

	Mean ± SD
	
"low p-estradiol" – 1. admission	0.31 ± 0.08 (0.23–0.43)
"high p-estradiol" – 2. admission	0.95 ± 0.25 (0.69–1.33)*

Values are mean ± SD. *Low/high: p < 0.001

### Correlations

We found no correlation between AQP-2 excretion and secretion of AVP or oxytocin, but a significant positive correlation between AQP-2 and urine osmolality (r = 0.3107, p = 0.0024) and reabsorption of free water (r = 0.3618, p < 0.001). At night these correlations were even stronger (0.5632, p = 0.02 and 0.6723, p = 0.004, respectively). AVP correlated negatively to diuresis (r = -0.3553, p = 0.004) and positively to urine osmolality (r = 0.2856, p = 0.02). The similar correlations with oxytocin were insignificant. Prolactin correlated with diuresis (r = -0.4240, p < 0.001) and urine osmolality (r = 0.2737, p = 0.03). There was a significant positive correlation between excretion of sodium and osmoles and diuresis (r = 0.6830, p < 0.001 and r = 0.5945, p < 0.001, respectively).

### Blood pressure

The measurements demonstrated a significant night time decrease in mean arterial blood pressure (MAP) of approximately 14% in both admissions (p < 0.001). There was no difference in MAP between the first and second admission.

## Discussion

We were able to demonstrate a circadian rhythm of AVP, ANP and aldosterone but not for oxytocin. The excretion of AQP-2 does not appear to be circadian, whereas PGE-2 has a marked nocturnal decrease in excretion. All hormones were unaffected by different concentrations of endogenous estrogen. Furthermore we showed a difference in p-osmolality between the mid-follicular (low estrogen) and the preovulatory phase (high estrogen). Plasma sodium and packed cell volume changed in parallel with plasma osmolality, suggesting a small increase in plasma volume due to high endogenous estrogen. Urine production did not change; neither did the renal excretory variables between the two admissions, even though there was a tendency towards sodium retention when estrogen was high.

To test the hypothesis that different endogenous levels estrogen alters the body fluid regulation and urine production, we designed this study with an admission in midfollicular phase (estrogen low) and an admission just before estimated time of ovulation (estrogen high). We selected the follicular phase, as the concentration of progesterone remains low in normal healthy subjects, throughout the study period.

Examining the effects of high and low estrogen on urine regulation and body fluid balance is complex in young natural cycling women because of the constantly changing concentration of sex hormones. Unfortunately seven of the included women in the present study had to be excluded prior to statistical handling due low concentration of estrogen in both admissions. A natural physiological variation in the length of the menstrual cycle may be an explanation.

A circadian rhythm in AVP is well-established. At least in males [4-6]. Only a few circadian studies of AVP have been conducted in females [8,17,18]. Former studies have been able to demonstrate a rise in both the basal concentration [7] and the magnitude of the nocturnal peak in AVP in follicular phase of the menstrual cycle compared to the luteal phase [8]. Other studies have failed to confirm changing basal AVP over the course of the menstrual cycle [9-12,17]. Exogenous estrogen unopposed to progesterone increases basal concentration of vasopressin, whereas progesterone in combination with estrogen lowers basal AVP-concentration [13-15]. Progesterone antagonizes the stimulatory effect of estradiol on vasopressin secretion [15] thereby reducing the nocturnal rise in AVP as well. In the present study we found identical plasma concentrations of AVP and identical nocturnal peaks in the two admissions. An explanation of this discrepant finding could be that we admitted solely in the follicular phase with similar levels of progesterone. Furthermore the variations in AVP concentrations could be related to the particular subtype of estrogen receptor activated. Young premenopausal women express mainly estrogen-receptor-β (ERβ), whereas postmenopausal women express estrogen-receptor-α (ERα)[31]. Since ERβ inhibits and ERα stimulates AVP neuronal activity in the supraoptic nucleus of the hypothalamus [32], this explains the elevated AVP levels in postmenopausal women in response to estrogen administration and the lack of increase in the present study.

Over the past 20 years several studies have shown that the operating point for AVP-release is shifted during the course of the menstrual cycle. Observational studies have shown a decrease in both plasma osmolality and sodium with no change in p-AVP in the luteal phase of the menstrual cycle [9,10] and in pregnancy [33]. Exogenous estrogen and progesterone or stimulation with gonadotropins result in a similar resetting [16,20]. Evidence favors the estrogenic component to be is responsible for the shift in osmoregulation [19,20]. We demonstrated a decrease in plasma osmolality and sodium of approximately 2.5 mosm/kg and 0.5 mmol/l, respectively. This equals only half the differences referred. This may be due to the different experimental set up. We focused on the effect of different concentration of estrogen unopposed to progesterone and have made our investigations solely in the follicular phase of the menstrual cycle. The greater difference found in previous studies may be due to the fact that they have admitted the women in early follicular and late luteal phase, thereby obtaining maximal difference in plasma-estradiol [9,10]. In young women estrogen administration will lower the osmotic threshold for AVP release, but no effect on free water reabsorption has been noted [19,21]. This has led to a suggestion that estrogen may lower the renal tubular sensitivity to AVP as well. Studies in rats show that estrogen inhibits AVP actions on free water reabsorption [34]. However, estrogen administration to young females does not affect the overall renal sensitivity to AVP and it even appears that estrogen increases the urine concentrating response within normal physiological ranges of AVP [35]. In the present study we did not observe any changes in reabsorption of free water between the two states of estradiol, nor did we demonstrate an increase in urine osmolality in high estrogen states, indicating that elevations in estradiol seen in the present study neither inhibits nor stimulates the AVP response. The fluid retention during estrogen treatment is therefore thought to be more related to increases in sodium reabsorption. In the present study, high estradiol – even though not quite significant – led to a strong tendency towards decreases in sodium excretion, fractional excretion of sodium and sodium clearance. This statistical significance is however influenced by an extreme sodium excretion in one of the participating women. Excluding this participant on behalf of these data would be incorrect, but a sensitivity analysis without her eliminates the statistical significant difference in sodium excretion. In young women administration of estradiol will increase sodium reabsorption during AVP infusion [35]. Estrogen administration have also led to greater sodium retention in response to hypertonic saline infusion [21]. We were not able to find significant sodium retention in the high-estradiol states, but several reasons could account from this discrepant finding. 1) Our study is purely observational; none of the physiological processes have been speeded up. 2) Plasma-estradiol may be insufficiently increased in the second admission. 3) Additionally the excretion of sodium during the in-patent investigations reflects the dietary intake of sodium at home as well. Participants were fluid preconditioned 2 days prior to the admissions, whereas sodium intake at home was not standardized. Perhaps because of differences in the participants' habitual sodium intake, the sodium intake during admission (3 mmol/kg/day) did not fully sodium load some of the participants while overload others.

In this study, we found no diurnal variation in concentration of oxytocin, but a variation in concentration between the two admissions. This significance is however partly a result of high values of oxytocin in one of the participating women. Excluding her form the primary analysis would eliminate the statistical significance, but still there would be a strong tendency towards an increased oxytocin concentration in the second admission. Previous studies have been able to demonstrate a fall in oxytocin during the day in healthy young males [5,6] and a significant nocturnal peak similar to the nocturnal peak in vasopressin release [5]. However, Kostoglou-Athanassiou et al [8] found in young women that p-oxytocin did not display diurnal variation, nor could they find any difference between the follicular and the luteal phase. Nevertheless other studies done during day-time only have revealed increased oxytocin concentrations at ovulation/midcycle [24,25].

Whether oxytocin in physiological doses plays a role in antidiuresis is elusive. In the present study there was no correlation between oxytocin and urine osmolality or diuresis, whereas the similar correlations with AVP were weak but significant. Together with the lack of nocturnal increase, this suggests that oxytocin in the present experimental set up do not contribute to the overall antidiuretic effect. In humans an antidiuretic effect of oxytocin has however been demonstrated [22,23]. The antidiuretic effect of oxytocin is believed to be mediated by the vasopressin receptor, since infusion of oxytocin in healthy young males produces an increase in AQP-2 excretion in parallel with its antidiuretic effect [22]. Since the affinity of oxytocin to the vasopressin receptor is much lower than that of AVP, the antidiuretic effect will probably only occur when supra-physiological doses of oxytocin are being used.

The urinary AQP-2 excretion did not display a circadian rhythm nor did it differ between the admissions. In DDAVP induced antidiuresis AQP-2 excretion rates correlate well with urine osmolality [36,37], whereas a correlation between the native AVP and AQP-2 excretion under different experimental conditions (dehydrating experiments or hypertonic saline infusion) is less obvious. Under normal physiological conditions we would expect the AQP-2 excretion to be higher during the night. The in-patient studies were designed to resemble the daily life of the participants and were purely observational. Examining the circadian rhythm in diuresis is very dependent on a normal sleep pattern. If sleep is interrupted diuresis will increase [38]. Blood sampling at night was carried out with care not to wake the participants, and this was possible in most cases. Since none of the healthy volunteers normally have nocturia or are awake at night, it would certainly interrupt their normal rhythm, if they were asked to empty their bladder at night or were asked to have a drink of water. Unfortunately this also entails, that the night time urine production is pooled in the morning and a possible increase in the excretion of AQP-2 at the time of maximal antidiuresis will be blurred. A urethral catheter could have been the solution, but the subsequent discomfort may disrupt the sleep and adversely affect the diurnal rhythm. Furthermore a catheter may interfere with the physiological processes of urine production and bladder filling.

Urine production at home averaged 10 ml/kg less than when the young women were admitted. Interestingly it was only 24 h and day time urine volume, whereas night time urine volume and day/night ratio were similar at home and during admissions. Fluid intakes were similar. Differences in extra renal fluid loss could explain this discrepant finding. In the hospital the volunteers were basically less active than at home. It's a question, of course, whether all voids were registered at home. The volunteers were motivated and thoroughly informed of the importance of a precise registration. All FVC schemes were examined carefully and no irregularities were detected. The diurnal profile in the hospital may be different from the profile at home, and the present data emphasize that fluid balance reports are influenced by the setting of the investigation.

## Conclusion

This study is to the best our knowledge the first to describe the diurnal urine regulation in women with focus on the different concentrations of endogenous estrogen. The study confirms the finding of a resetting of the osmoreceptors in high-estrogen states. We found that the hormones involved in the diurnal urine regulation was unaffected by the level of estradiol. Furthermore it appears that oxytocin under normal physiological conditions has no influence of the overall antidiuretic effect. This study has however certain obvious limitations. Due to the large irregularity of a normal regular menstrual cycle only half of the participating women were actually included in the statistical analysis. Furthermore it is possible that the difference obtained in the concentration of p-estradiol between the first and second admission is too small to actually alter the diurnal water homeostasis. More studies of the influence of estrogen on the diurnal urine regulation under normal physiological conditions are therefore warranted.

## Competing interests

The authors declare that they have no competing interests.

## Authors' contributions

CGJ, GMH and JCD conceived the idea and designed the study. CGJ conducted the data collection and the analysis of data. JF conducted the AQP-2 and PGE-2 analyses. PB conducted the analysis of oxytocin. All authors contributed to interpretation of data and provided significant input for bringing the message through in the manuscript. All authors contributed to the interpretation of the data and the final preparation of the paper. All authors have read and approved the final manuscript.

## Pre-publication history

The pre-publication history for this paper can be accessed here:


